# Endovascular Retrieval of a Detached and Dislocated Venous Port Catheter in the Right Heart Chamber Using a Triple-Loop Snare Device

**DOI:** 10.7759/cureus.33681

**Published:** 2023-01-12

**Authors:** Nikolaos Papatheodorou, Christos Argyriou, Sotirios Botaitis, Dimitrios E Diamantidis, Georgios Georgiadis

**Affiliations:** 1 General Surgery Department, Democritus University of Thrace, Alexandroupolis, GRC; 2 Vascular Surgery Department, Democritus University of Thrace, Alexandroupolis, GRC; 3 1st General Surgery Department, University Hospital of Alexandroupolis, Alexandroupolis, GRC

**Keywords:** port catheter, migration, fracture, detachment, complication

## Abstract

The authors present a case of a successful percutaneous retrieval of a detached port-a-catheter device that had migrated to the right cardiac chambers in a patient with inoperable pancreatic cancer and hepatic metastases. The patient was admitted to the vascular clinic department on an urgent basis due to an accidental detachment of the catheter during removal at another hospital. The catheter had migrated from the initial placement site in the right subclavian vein to the superior vena cava and right heart chambers. Under local anesthesia, the right femoral vein was accessed using the Seldinger technique, and the migrated catheter was retrieved using a triple-snare-loop device for foreign body removal. Chest radiography after the retrieval procedure did not show any foreign bodies in the right heart chambers or superior vena cava. The patient was discharged home the following day.

## Introduction

The implantation of these devices is recommended for patients who need long-term intravenous therapy, particularly in cancer patients who are frail and require the delivery of chemotherapeutic-cytotoxic drugs (administered in solutions with high osmolality) [1,2]. These drugs have a well-known sclerosing effect on peripheral vessels, and the use of a venous port catheter can reduce the need for frequent blood sampling [1,3].

Complications of port catheters are not uncommon and can be divided into early (occurring within 30 days of implantation) and delayed (occurring more than 30 days after implantation) complications. Early complications include pneumothorax, hemothorax, and catheter malposition while delayed complications include infection, thrombosis, catheter fracture with extravasation and migration, and distal embolization [4]. Fracture and migration of a port catheter is a rare complication that can occur after long-term access to the central venous system. If this occurs, the device may migrate and become dislodged in the superior vena cava, right atrium and ventricle, inferior vena cava, or pulmonary artery [5,6].

## Case presentation

**Figure 1:**
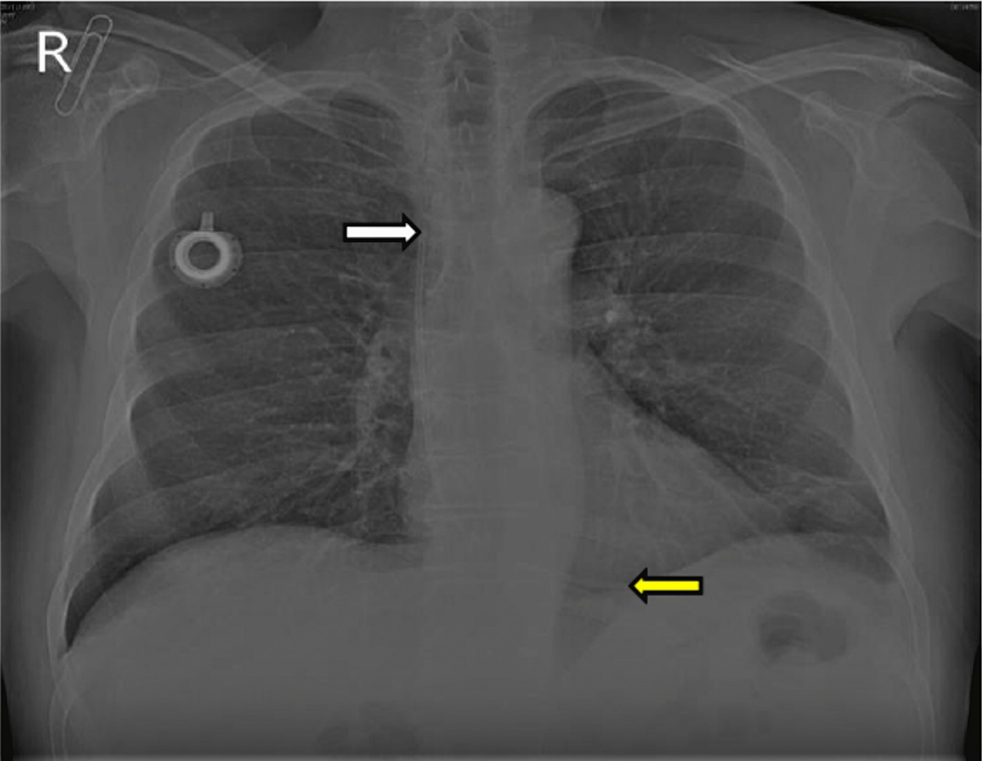
Chest radiography showing the proximal tip of the catheter fragment (white arrow) lodged at the superior vena cava and the distal tip (yellow arrow) at the right ventricle

A 58-year-old male patient with a history of pancreatic cancer and a central venous port-a-catheter for chemotherapy was admitted to our institution after the catheter was accidentally detached and migrated during removal. Upon admission, the patient was asymptomatic, but chest radiography showed that the disconnected catheter had its proximal end located in the superior vena cava and its distal end in the right ventricle (Figure 1).

**Figure 2:**
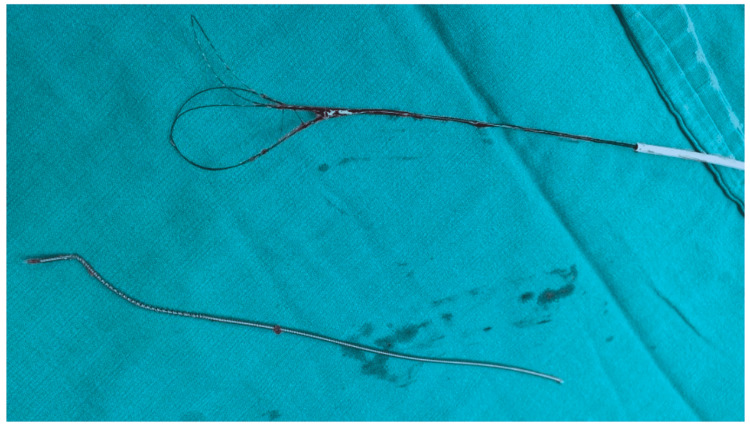
The retrieved fractured catheter, along with the three-loop-snare catheter used for the retrieval procedure

Given the potential risk of further migration and associated fatal complications, it was determined that prompt retrieval of the catheter was necessary. The patient provided written informed consent for a percutaneous transcatheter retrieval procedure. Under locoregional anesthesia, the Seldinger technique was employed to place a 12 French femoral sheath in the right femoral vein, and a hydrophilic guidewire was inserted into the inferior vena cava. A triple-looped snare catheter was advanced through the sheath to the lodged site, and the snare was used to capture the tip of the catheter fragment and was then carefully withdrawn through the right femoral vein and externalized (Figure 2).

**Figure 3:**
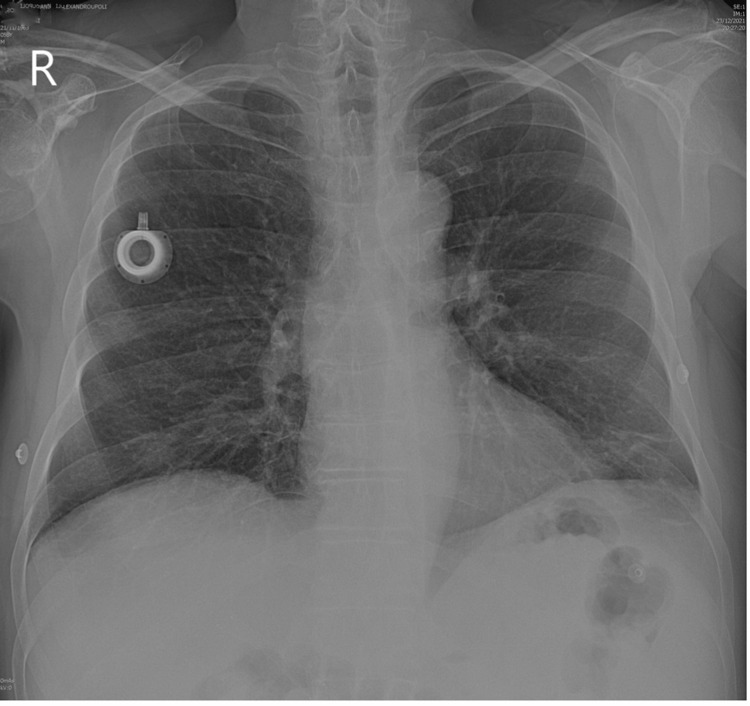
Postoperative chest radiography showing the complete retrieval of the catheter fragment

The total fluoroscopy time was seven minutes. The patient was continuously monitored throughout the procedure, and a postoperative chest radiography was obtained to confirm the successful retrieval of the migrated catheter (Figure 3).

No complications were observed during or after the retrieval procedure. The patient's postoperative course was uncomplicated.

## Discussion

Complications of port catheters may arise during the implantation procedure or maintenance period. These complications can be divided into infectious, with an incidence ranging from 0.6% to 27%, and non-infectious complications [4]. Non-infectious complications with a much lower incidence include malfunction, extrusion of the reservoir, thrombosis (the second most common complication after infection, with bacteremia being the most common), detachment of the catheter, fracture, and migration with or without embolization [7]. Catheter fracture and migration during the maintenance period are rare, with an incidence of less than 0.4% [1]. These complications may result in further damage to the heart muscle, such as perforation, ventricular rupture, cardiac tamponade, pseudoaneurysm, and thrombosis, and may also cause heart rhythm disorders [4,5,8].

Risk factors for catheter fracture and migration include external compression from tight clothing or seat belts, increased intracatheter pressure during repetitive flushing with the use of small (5 mL) syringes, loose connection between the reservoir and the catheter, and material fatigue due to the formation of a sharp angle during the implantation procedure [1,5]. Moreover, another risk factor is the pinch-off syndrome, which is more common in port-a-catheters placed via the subclavian vein due to compression of the catheter between the clavicle and the first rib [5,8,9]. The incidence of catheter fracture due to the pinch-off syndrome is reported to be 1.1% to 5% [9], whereas the incidence of catheter disconnection is 3.5% [10].

Central venous port-a-catheters should be heparinized regularly, using a 10 mL syringe immediately after each use or monthly in case of inactivity [1]. Regular chest radiography is recommended to determine the proper position of the catheter and any complications that may arise [2]. Digital subtraction angiography with contrast medium injection is the gold standard for diagnosing port-a-catheter complications [6]. It has a high diagnostic performance and allows for potential intervention through an endovascular approach. Computed tomography angiography may be an alternative, but it is a time-consuming procedure without the opportunity for potential intervention [6].

Fragmented catheter positioning can vary and may be located in the hepatic vein, brachiocephalic vein, superior vena cava, pulmonary artery, right atrium, right ventricle, or inferior vena cava [5,6,8,11]. The most common dislodgment sites are the right atrium, pulmonary artery, superior vena cava, and right ventricle [11].

Symptoms associated with port catheter disconnection, fracture, and migration include local pain, swelling (subcutaneous extravasation), and neck bulging during port flushing [9]. It is necessary to consider the risk of catheter-associated thrombosis in the presence of these symptoms [10].

The removal of the fragmented particles should be imminent because its permanence over six to eight weeks is associated with an increased risk of endothelial reaction and fibrosis, vascular wall perforation, arrhythmias, and sudden death [5].

The management of such complications may involve the surgical (open thoracotomy) or endovascular approach. Some authors recommend expectant management with anticoagulation until symptoms appear [5]. The gold standard for intravascular foreign body removal is the percutaneous endovascular retrieval procedure, which uses tools such as triple-loop-snares, retrieval baskets, gooseneck traps, and grasping forceps while others support the use of the interlacing and traction technique with pigtail catheter [5].

## Conclusions

Catheter fracture and migration can pose a challenge for vascular surgeons and may require a multidisciplinary approach to prevent further life-threatening complications. There are currently no clear guidelines for the diagnostic and therapeutic management of port catheter fracture and migration. Therefore, it is essential to promptly recognize and treat these complications.
